# Supplementary figures and images for: Production of Soda Lime Glass Having Antibacterial Property for Industrial Applications
Source: Materials (Basel). 2020 Oct 28;13(21):4827. doi: 10.3390/ma13214827 (PMC7663106; doi:10.3390/ma13214827)

## Slide 1
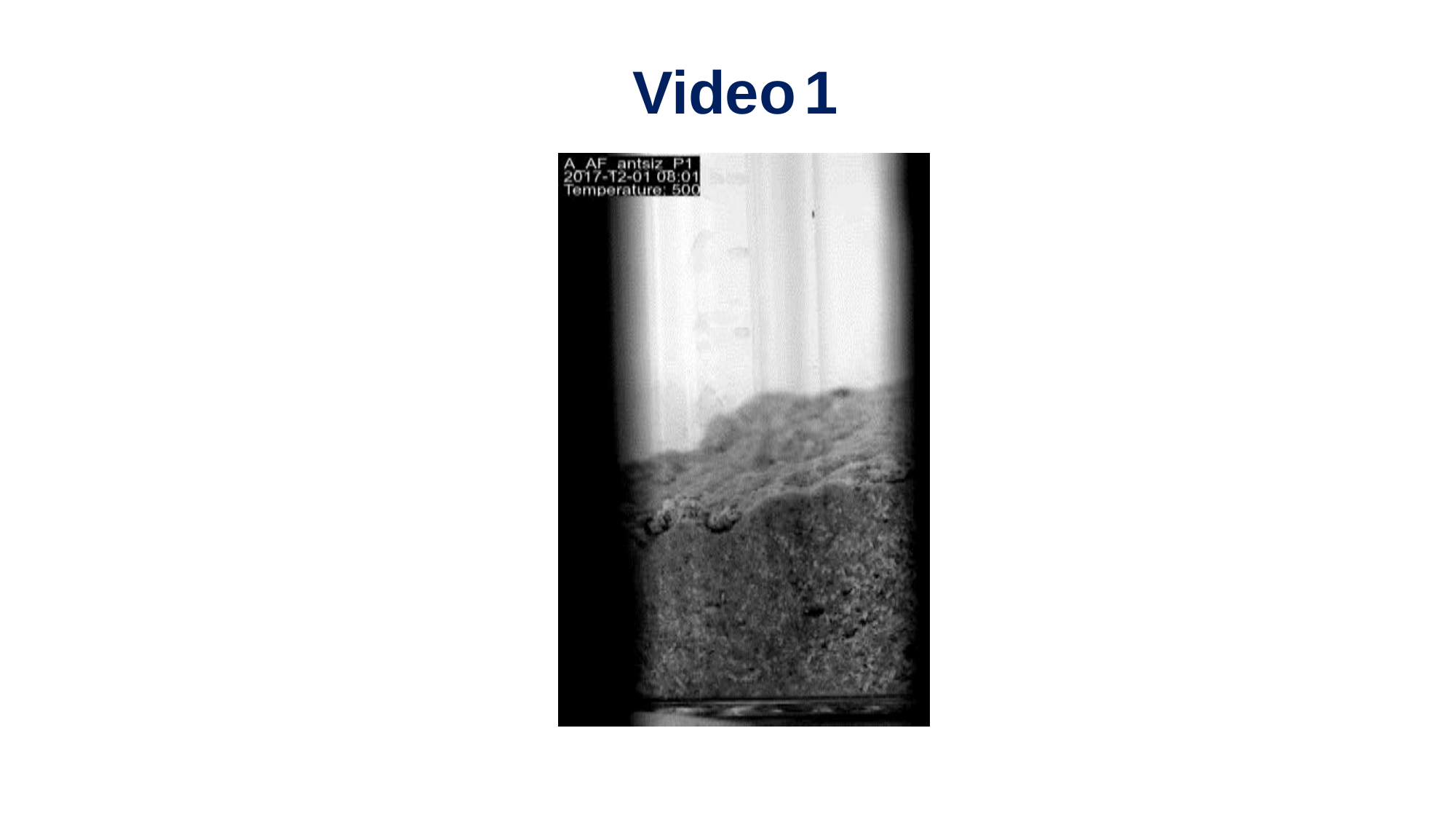

Video 1

Supplement: Supplementary file 1 [file materials-13-04827-s001.zip › Videos/Video 1.pptx]

## Slide 1
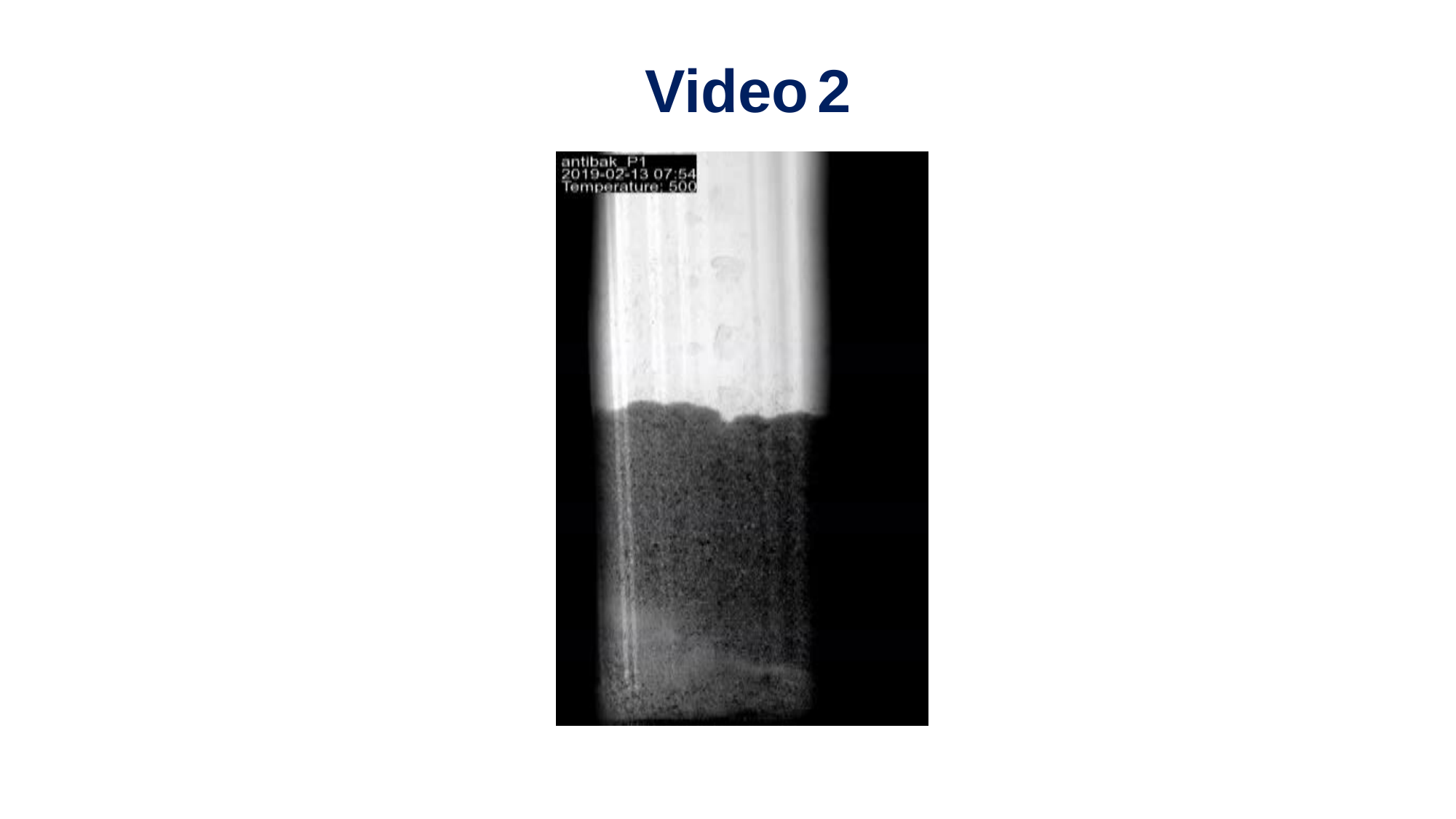

Video 2

Supplement: Supplementary file 1 [file materials-13-04827-s001.zip › Videos/Video 2.pptx]
